# Release of Bisphenol A from Milled and 3D-Printed Dental Polycarbonate Materials

**DOI:** 10.3390/ma14195868

**Published:** 2021-10-07

**Authors:** Antonin Tichy, Marketa Simkova, Josef Schweiger, Pavel Bradna, Jan-Frederik Güth

**Affiliations:** 1 Institute of Dental Medicine, First Faculty of Medicine, Charles University, General University Hospital, Karlovo Namesti 32, 121 11 Prague, Czech Republic; pavel.bradna@lf1.cuni.cz; 2 Institute of Endocrinology, Narodni 139/8, 116 94 Prague, Czech Republic; msimkova@endo.cz; 3 Department of Prosthetic Dentistry, University Hospital, LMU Munich, Goethestraße 70, 80336 Munich, Germany; josef.schweiger@med.uni-muenchen.de; 4 Department of Prosthodontics, Center for Dentistry and Oral Medicine (Carolinum), Goethe University Frankfurt, Theodor-Stern-Kai 7, 60596 Frankfurt, Germany; gueth@med.uni-frankfurt.de

**Keywords:** polycarbonate, dental prosthesis, splint, bisphenol A, milling, 3D-printing, chromatography, mass spectrometry

## Abstract

Polycarbonates are polymers of bisphenol A (BPA), a well-known endocrine disruptor. This study evaluated the release of BPA from polycarbonate crowns that were (1) milled from Temp Premium Flexible (ZPF, Zirkonzahn, Italy) or Tizian Blank Polycarbonate (TBP, Schütz Dental, Germany), or (2) 3D-printed (Makrolon 2805, Covestro, Germany). Commercial prefabricated polycarbonate crowns (3M, USA) and milled poly(methyl methacrylate) (PMMA) crowns (Temp Basic, Zirkonzahn, Italy) were included for comparison. The crowns were stored at 37 °C in artificial saliva (AS) or methanol, which represented the worst-case scenario of BPA release. Extracts were collected after 1 day, 1 week, 1 month and 3 months. BPA concentrations were measured using liquid chromatography-tandem mass spectrometry. The amounts of released BPA were expressed in micrograms per gram of material (μg/g). After 1 day, the highest amounts of BPA were measured from milled polycarbonates, TBP (methanol: 32.2 ± 3.8 μg/g, AS: 7.1 ± 0.9 μg/g) and ZPF (methanol 22.8 ± 7.7 μg/g, AS: 0.3 ± 0.03 μg/g), followed by 3D-printed crowns (methanol: 11.1 ± 2.3 μg/g, AS: 0.1 ± 0.1 μg/g) and prefabricated crowns (methanol: 8.0 ± 1.6 μg/g, AS: 0.07 ± 0.02 μg/g). Between 1 week and 3 months, the average daily release of BPA in methanol and AS decreased below 2 μg/g and 0.6 μg/g, respectively. No BPA was released from PMMA in AS, and the cumulative amount released in methanol was 0.2 ± 0.06 μg/g. In conclusion, polycarbonates could be a relevant source of BPA, but the current tolerable daily intake of BPA (4 μg/kg body weight) should not be exceeded.

## 1. Introduction

Bisphenol A (BPA; 2,2-bis(4-hydroxyphenyl) propane) is an endocrine disruptor [1], affecting the hormonal system through the interaction of its phenolic structure with various receptors [2]. As a consequence, BPA is associated with various disorders, mainly reproductive, developmental and metabolic [3]. However, since BPA may act either as a receptor agonist or antagonist under different circumstances, its dose–response relationship is nonmonotonic, and the effect on human health is therefore difficult to predict [1,4]. Some researchers even emphasize a persisting health risk at doses lower than the current tolerable daily intake (TDI) of 4 μg/kg body weight (b.w.) set by the European Food Safety Authority (EFSA) in 2015 [5].

Concerns about the release of BPA from dental materials were raised after Olea et al. reported the estrogenic activity of a dental sealant containing bisphenol A dimethacrylate (BisDMA) and considerable levels of BPA in the saliva of patients treated with the sealant [6]. Despite the controversy over these results [7,8], the release of BPA from dental sealants and resin-based composites was confirmed in numerous studies since then [9,10]. The amounts of released BPA were inconsistent, but generally significantly below TDI, which led the European Commission’s independent Scientific Committee on Emerging and Newly Identified Health Risks (SCENIHR) to conclude that long-term oral exposure to BPA via dental materials poses only a negligible risk to human health [11].

Various dental resin-based materials contain monomers derived from BPA, but free BPA is present only in trace amounts as a contaminant or a degradation product of the monomers [9,12,13,14]. In contrast, BPA is the key building block of polycarbonates that are used in dentistry as orthodontic brackets, denture base resins, prefabricated temporary crowns and splints. Although the potential of polycarbonates to release BPA in the oral environment might be higher compared to dental sealants and resin-based composites, it has not been thoroughly examined. Suzuki et al. reported that the amounts of BPA released from polycarbonate orthodontic brackets and denture base resins after 1 h were 0.01–0.04 μg per gram of material (μg/g) in water and 0.12–9.42 μg/g in ethanol [15]. The released amounts increased significantly if the materials were crushed into powder or heated during denture manufacturing [15]. Watanabe et al. [16] found that the release of BPA from orthodontic brackets in water was significantly affected by temperature, as the release at 60 °C was approximately 28-fold higher than at 37 °C. However, it was concluded that the amounts of released BPA should have little or no estrogenic effect in practice [16]. In another study, it was revealed that the content of BPA in dental polycarbonate appliances increased during storage in water, indicating their hydrolytic degradation [17].

Recently, polycarbonate splints manufactured using the computer-aided design/computer-aided manufacturing (CAD/CAM) technology were introduced for the functional and esthetic evaluation of newly defined occlusal dimensions [18]. Owing to the high strength, toughness and durability, very thin polycarbonate splints can be fabricated. Furthermore, their esthetic appearance favorably affects patient compliance compared to poly(methyl methacrylate) (PMMA) splints [18]. On the other hand, the splints could release considerable amounts of BPA, given their large surface area. To assess the risk, this study measured the release of BPA from milled and 3D-printed crowns representative of occlusal splints in artificial saliva and methanol. Commercial prefabricated polycarbonate crowns and milled PMMA crowns were tested for comparison. Extracts were collected at several time points (1 day–3 months) to determine the kinetics of BPA release. In addition, the sorption and amount of extractable matter in artificial saliva were measured, and scanning electron microscopy was used for the observation of crown surface morphology. The null hypotheses were that there would be no difference (1) between the amounts of BPA released in artificial saliva and methanol, and (2) in the daily release of BPA at the tested time points.

## 2. Materials and Methods

**Figure 1:**
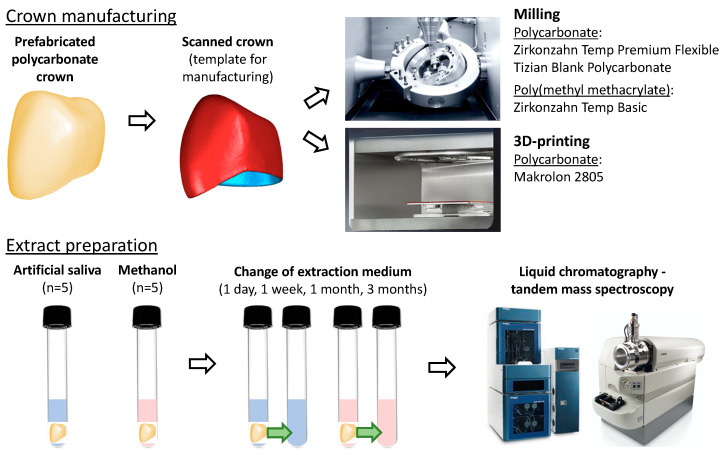
A schematic diagram of the experimental procedures.

The polycarbonate materials included prefabricated polycarbonate crowns-mandibular first premolars (lot number NC00297; 3M, St. Paul, MN, USA), crowns milled from Zirkonzahn Temp Premium Flexible shade A3-B3 (ZPF; lot number 11714; Zirkonzahn, Gais, Italy) and Tizian Blank Polycarbonate shade A2 (TBP; lot number 2020001641; Schütz Dental, Rosbach, Germany), and crowns 3D-printed from Makrolon 2805 (Covestro, Leverkusen, Germany). PMMA crowns were milled from Zirkonzahn Temp Basic shade A3-B3 (lot number 6795; Zirkonzahn). There were ten crowns per group. The experimental procedure is illustrated in Figure 1.

### 2.1. Crown Preparation

**Figure 2:**
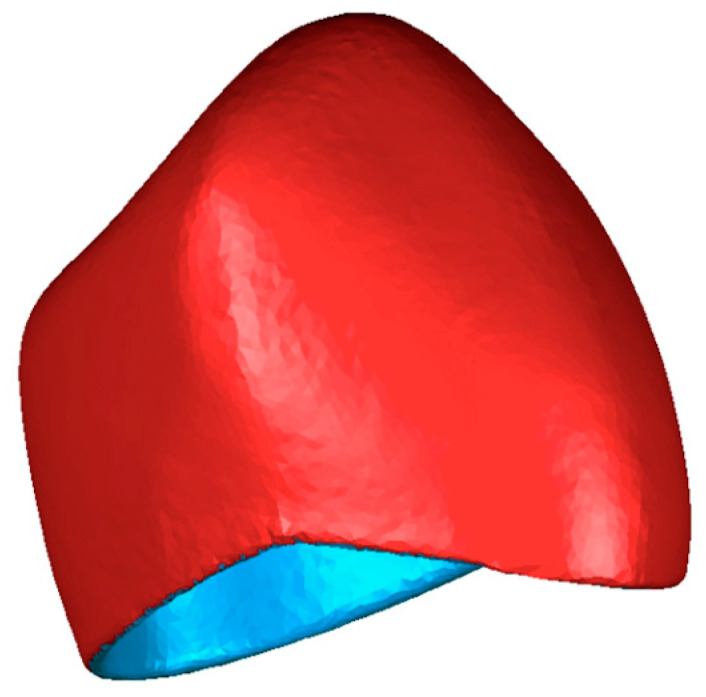
The STL file of the scanned prefabricated polycarbonate crown, which served as a template for CNC manufacturing.

To standardize specimen dimensions, a prefabricated polycarbonate crown was scanned using the S900 Arti scanner (software ZirkonzahnScan v.5051.3; Zirkonzahn) and used as a template for milling and 3D-printing (Figure 2). The surface area of the crowns was 310.9 mm^2^, and their volume equaled 108.9 mm^3^ (Geomagic Qualify 2012, Morrisville, NC, USA). The exported STL dataset was digitally nested in the milling blanks. ZPF and PMMA crowns were milled using the M1 computer numerical control (CNC) milling machine (software Zirkozahn.Fräsen v.04_4002_0030; Zirkonzahn) in “High Quality” mode using the dry milling technique. Rough milling was performed using the 2 L Premium bur at 12,000 rpm, followed by precise milling using the 1 L bur at 10,000 rpm and the 0.5 S bur at 13,000 rpm. TBP crowns were milled using the Motion 2 CNC milling machine (Amann Girrbach, Koblach, Austria) in the highest quality using the wet milling mode. Rough milling was performed using the Roto RFID 2.5 PMMA bur at 15,000 rpm, followed by the Roto RFID 1.0 PMMA bur at 15,000 rpm and the Roto RFID 0.6 PMMA bur at 18,000 rpm. After milling, the crowns were separated from the milling discs using a cross-cut tungsten carbide cutter (number H257EF.104.023; Komet, Lemgo, Germany). 3D-printed crowns were prepared from Makrolon 2805 using the “drop-on-demand” technology in the Freeformer 200 3D printer (Arburg, Lossburg, Germany). The procedures were performed according to the respective manufacturer’s recommendations.

The outer surface of the manufactured crowns was smoothed using a disc-shaped silicone polisher (universal polisher 9627.900.220; Komet), followed by pre-polishing of the crowns with the technician’s handpiece, a goat hairbrush, and Acrypol polishing paste (Bredent, Senden, Germany). Polishing to high gloss was performed using a polishing motor equipped with a wool swab and Abraso Starglanz polishing paste (Bredent). The crowns were cleaned using the Wasi-Steam 2 steam jet unit (Wassermann Dental-Maschinen, Hamburg, Germany), followed by ultrasonic cleaning in distilled water (Sonorex Super; Bandelin, Berlin, Germany) for 5 min. Finally, the specimens were dried using oil-free compressed air and left to dry at room temperature for at least 4 weeks.

### 2.2. Extract Preparation

The crowns were weighed using a digital analytical balance accurate to 0.1 mg and transferred to glass test tubes containing either 2 mL of LC-MS grade methanol or artificial saliva (*n* = 5). The artificial saliva (Hospital laboratory; General University Hospital in Prague, Czech Republic) was prepared by dissolving 0.8 g/L NaCl, 1.2 g/L KCl, 0.1 g/L CaCl_2_·2H_2_O, 0.3 g/L K_2_HPO_4_·3H_2_O, and 0.1 g/L MgCl_2_·6H_2_O in distilled water [19] with pH adjusted to 7.0. The test tubes were closed using screw caps with septa covered with a layer of polytetrafluoroethylene and incubated at 37 °C. Methanol or the artificial saliva was changed after 1 day, 1 week, 1 month (28 days), and 3 months (84 days) to determine the kinetics of BPA release. After extracts had been transferred into new glass test tubes, the crowns were carefully removed, gently air-dried until their surface was visibly free of moisture, weighed, and re-placed inside of the original test tubes, which had been rinsed with 0.5 mL of methanol five times to eliminate any remnants of BPA. Then, 2 mL of methanol or the artificial saliva were added, and the test tubes were placed in the incubator. To avoid contamination, only glass and metal instruments that had been repeatedly cleansed with methanol were used.

### 2.3. Liquid Chromatography-Tandem Mass Spectrometry (LC-MS/MS)

BPA and deuterated BPA (d16BPA) standards, acetone, sodium bicarbonate, ammonium formate and dansyl chloride were purchased from Sigma-Aldrich (St. Louis, MO, USA). Diethylether, LC-MS grade methanol and water for chromatography were purchased from Merck (Darmstadt, Germany). Methanol p.a. was purchased from Lach-Ner (Neratovice, Czech Republic).

A stock solution of BPA in methanol was used to prepare calibration mixtures, using which a nine-point calibration curve (0.065–16.0 ng/mL) was constructed. Based on a pilot study, 10 µL of methanol extracts and 20 µL of artificial saliva extracts were analyzed for polycarbonates to fit the calibration range, whereas 500 µL were used for PMMA. The artificial saliva extracts were extracted using diethylether, methanol extracts were evaporated to dryness under reduced pressure. The dry residues and control samples with a known addition of BPA were then spiked with 10 µL of the internal standard (d16BPA in methanol) and evaporated to dryness again. The derivatization reaction was conducted according to [20,21,22]. In brief, 50 µL of dansyl chloride in acetone (1 mg/mL) and 50 µL of 100 mM sodium bicarbonate buffer were added to the dry residues and vortexed. After incubation at 50 °C for 15 min and evaporation to dryness, equal amounts of methanol and a 10 mM aqueous solution of ammonium formate were added. Then, 50 µL of the solution were injected and analyzed using API 3200 (Sciex, Concord, Canada), a triple-stage quadrupole mass spectrometer with electrospray ionization (ESI) connected to the Eksigent ultra LC 110 ultra-high performance liquid chromatography (UPLC) system (Redwood City, CA, USA). Chromatographic separation was performed using a Kinetex C18 1.7 µm (150 × 3.0 mm) column (Phenomenex, Torrance, CA, USA) equipped with a security guard at a flow rate of 0.4 mL/min at 50 °C. A mixture of methanol and water was used as the mobile phase. Further information about LC-MS/MS conditions is available in the referenced studies [20,21]. The lower limit of BPA quantification (LLOQ) was 0.042 ng/mL.

### 2.4. Sorption and Amount of Extractable Matter in the Artificial Saliva

After 3 months of immersion in the artificial saliva, the crowns were gently air-dried to remove moisture from their surface and weighed using the analytical balance. They were then left at room temperature to dry and weighed regularly until constant mass was obtained. Based on the mass prior to immersion (*m*_1_), the mass after the 3-month immersion (*m*_2_) and the mass after drying (*m*_3_), the artificial saliva sorption (*AS_sp_*) and amount of extractable matter (*AS_em_*) of the materials relative to the volume of the crowns (*V*) were calculated using following equations:

(1) $$AS_{sp}=\left(m_{2}-m_{3}\right)/V$$

(2) $$AS_{em}=(m_{1}-m_{3})/V$$

### 2.5. Morphological Analysis

Following immersion in the extraction media, the surface morphology of the crowns was observed using the SZX10 stereo microscope (Olympus, Tokyo, Japan) and the JSM 5500-LV scanning electron microscope (Jeol, Tokyo, Japan). After the initial examination of the polished outer surfaces, the crowns were cut into three pieces (occlusal surface, mesial wall, distal wall) and their unaltered structural characteristics were observed on the inner surfaces. Prior to the observation at various magnification, the crowns were sputter-coated with gold (JFC-1200 Fine Coater, Jeol, Tokyo, Japan).

### 2.6. Statistical Analysis

The analyses were performed in the R environment [23]. The amount of BPA in each extract was divided by the specimen mass (*m*_1_) and the extraction time in days to obtain the average daily release of BPA per gram of material. The data were skewed to the right, and they were therefore log-transformed. Since four extracts were prepared from each crown, a linear mixed-effect model was employed to take the random effect of individual crowns into account. In the analyses of BPA release, fixed effects of material, extraction medium, and immersion time were investigated. In the analyses of the artificial saliva sorption and amount of extractable matter, the fixed effect of the material was tested. Multiple comparisons were performed using Tukey’s post hoc test. The significance level was set to 0.05.

## 3. Results

### 3.1. BPA Release

BPA was detected in all extracts of polycarbonates, whereas PMMA released detectable amounts of BPA only in methanol during the first week (Table 1). Significantly more BPA was released in methanol than in the artificial saliva (*p* < 0.001) in all groups where BPA was detected. The average daily release of BPA was highest after 1 day, followed by a significant decrease from 1 day to 1 week (*p* < 0.001) and from 1 week to 1 month (*p* < 0.001) when the release reached its minimum. Compared to the values after 1 month, the average daily release of BPA after 3 months increased in all groups, significantly for prefabricated crowns and TBP in methanol and for ZPF in the artificial saliva.

**Figure 3:**
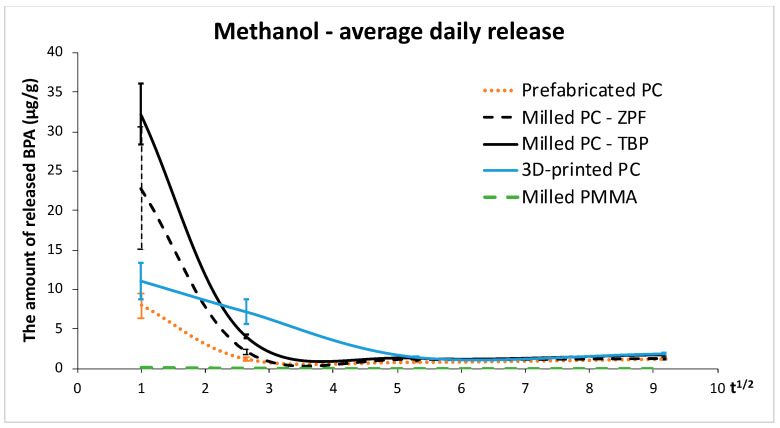
Average daily release of BPA in methanol (μg/g). The values are plotted against the square root of time, assuming that the release of BPA follows Fick’s law of diffusion. Abbreviations: PC—polycarbonate, ZPF—Zirkonzahn Temp Premium Flexible, TBP—Tizian Blank Polycarbonate, PMMA—poly(methyl methacrylate) (Zirkonzahn Temp Basic).

In methanol, the release of BPA was initially highest from milled polycarbonates (TBP and ZPF) (*p* < 0.001), while 3D-printed polycarbonate crowns released the highest amounts of BPA at the remaining time points. This is illustrated in Figure 3, which shows a steep decrease in the average daily release of BPA from all polycarbonates except for 3D-printed crowns whose release did not decrease as much. Prefabricated crowns released the least BPA of the tested polycarbonates at all time points, although the release after 1 month and 3 months was not significantly different from ZPF (*p* > 0.05). Trace amounts of BPA were found in the extracts of PMMA after 1 day and 1 week, and no BPA was detected after 1 month and 3 months, i.e., the values were below LLOQ.

**Figure 4:**
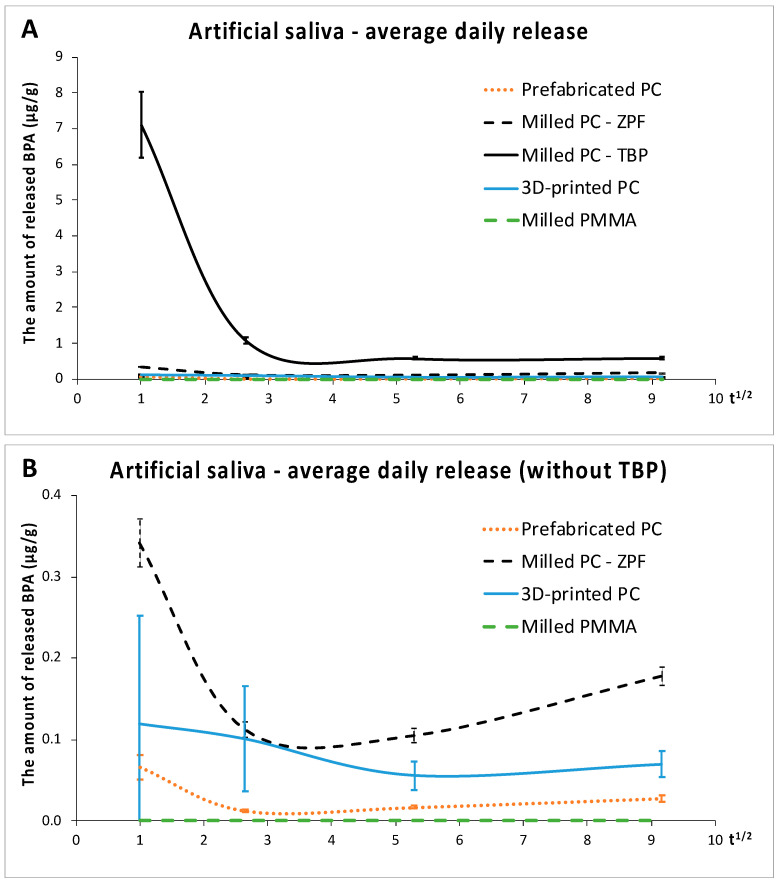
Average daily release of BPA in the artificial saliva (μg/g). (**A**): comparison of all materials, (**B**): comparison of materials without Tizian Blank Polycarbonate. Abbreviations as in Figure 3.

In the artificial saliva, the release of BPA from TBP was highest (*p* < 0.001), which is demonstrated in Figure 4A. Compared to the other milled polycarbonate (ZPF), which released the second highest amounts of BPA, the release from TBP was twentyfold after 1 day, tenfold after 1 week, sixfold after 1 month, and threefold after 3 months. Figure 4B shows that the average daily release of BPA from all polycarbonates decreased significantly between 1 day and 1 week (*p* < 0.001), except for 3D-printed crowns (*p* > 0.05). Like in methanol, prefabricated crowns released the least BPA of all polycarbonates (*p* < 0.01). Despite the low LLOQ and the use of a higher volume of the extract for the LC-MS/MS analysis, no detectable levels of BPA were found in the extracts of PMMA.

### 3.2. Sorption and Amount of Extractable Matter

The effect of material was significant for both the sorption and amount of extractable matter in the artificial saliva (*p* < 0.001), Table 2. The lowest sorption values were measured for milled polycarbonates, followed by prefabricated crowns. Overall, 3D-printed crowns exhibited the highest sorption among polycarbonates (*p* < 0.001), and its value did not differ significantly from the sorption of PMMA (*p* > 0.05). There was no significant difference in the amount of extractable matter between the materials except for 3D-printed crowns, which exhibited a significantly lower value than ZPF (*p* = 0.02).

### 3.3. Morphological Analysis

**Figure 5:**
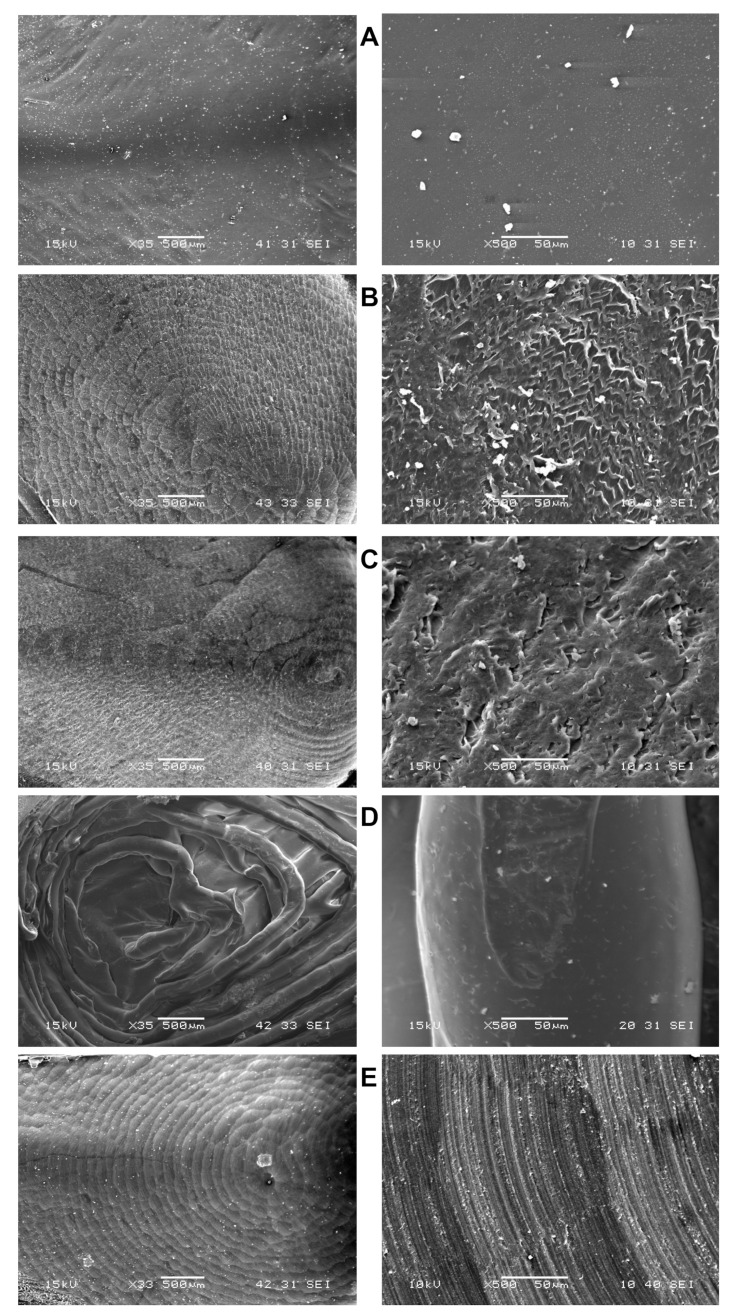
Morphology of the crowns’ inner surfaces. (**A**): Prefabricated polycarbonate crown; (**B**): Milled polycarbonate crown (ZPF); (**C**): Milled polycarbonate crown (TBP); (**D**): 3D-printed polycarbonate crown; (**E**): Milled PMMA crown. The surfaces of prefabricated crowns were smooth. Concentric patterns were observed in the occlusal parts of CNC-manufactured crowns at magnification 35× (left column). In milling, these patterns are called scallops and are caused by stepover, i.e., the space between passes of ball nose end mills. At magnification 500× (right column), plastically deformed zones and small cracks were observed in milled polycarbonates, especially ZPF, and shallow parallel grooves were present on the surfaces of PMMA crowns.

**Figure 6:**
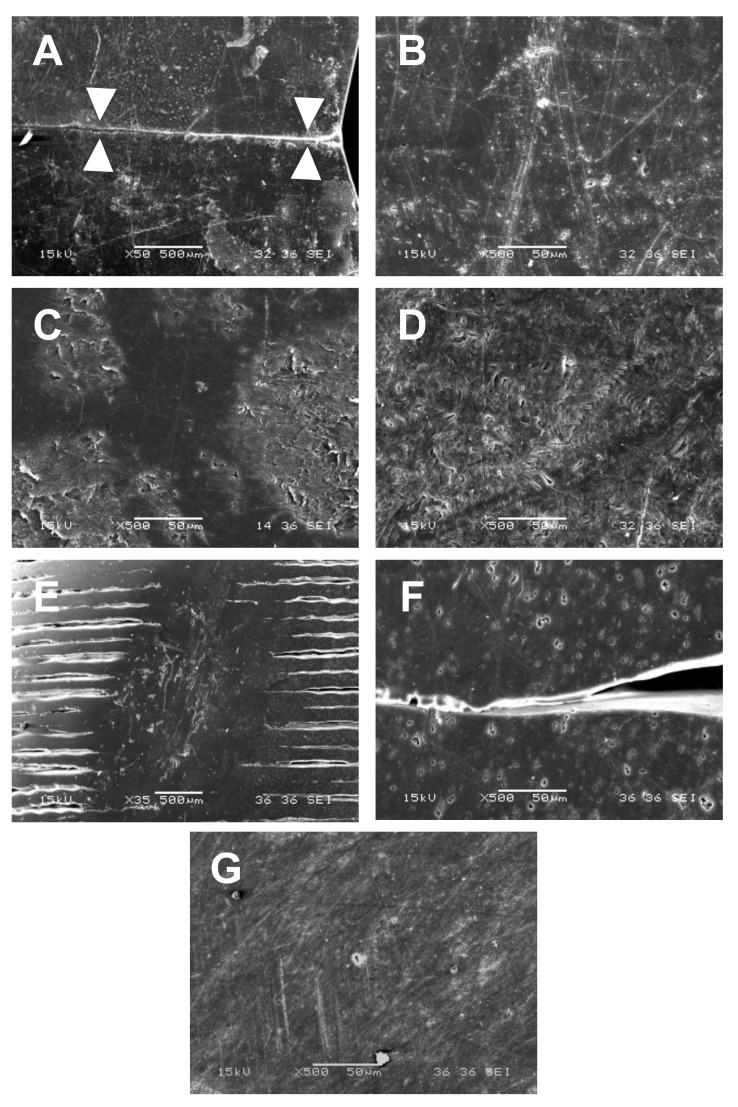
Morphology of the crowns’ outer surfaces. Magnification 50× (**A**,**E**) and 500× (**B**–**D**,**F**,**G**). (**A**,**B**): Prefabricated polycarbonate crown; (**C**): Milled polycarbonate crown (ZPF); (**D**): Milled polycarbonate crown (TBP); (**E**,**F**): 3D-printed polycarbonate crown; (**G**): Milled PMMA crown. White triangles in “A” point at the parting line. Despite polishing, irregularities and minor defects were observed on the surfaces of all crowns. Gaps between the filaments were observed on outer surfaces of 3D-printed crowns. The surfaces of PMMA crowns were smoother compared to polycarbonate crowns.

Prefabricated crowns had smooth inner surfaces (Figure 5A), but minor defects were present on outer surfaces and a parting line passing through the vestibular cusp was observed on the mesial and distal wall (Figure 6A,B). The inner surfaces of milled crowns were rougher, with parallel patterns on the crown walls and concentric patterns in the occlusal part. These patterns are called scallops and are caused by stepover, i.e., the space between passes of ball nose end mills (Figure 5B,C,E). At higher magnification, milled polycarbonates (Figure 5B,C) exhibited various surface irregularities; plastically deformed zones and small cracks were observed, especially on the inner surfaces of ZPF (Figure 5B). Despite polishing, the irregularities were noticeable even on the outer surfaces of ZPF (Figure 6C) and TBP (Figure 6D). Shallow parallel grooves without cracks were found on the inner surfaces of milled PMMA (Figure 5E), and outer surfaces were smoother than those of milled polycarbonates (Figure 6G). The observation of 3D-printed crowns revealed a layered structure resulting from the sequential deposition of the 3D printing filament (Figure 5D). Gaps between the filaments were observed even on the polished outer surfaces, together with minor voids within the filaments (Figure 6E,F).

## 4. Discussion

Despite concerns over the release of BPA from dental materials, little attention has been paid to polycarbonates, which are polymers of BPA linked with carbonate groups. Polycarbonates serve as an alternative to PMMA for the manufacturing of provisional crowns and occlusal splints, so the lack of attention can be attributed to their use in fewer indications compared to other resin-based restorative materials. The main advantage of polycarbonates over PMMA is that their strength and fracture toughness is higher [18]. Thanks to the increased impact resistance, they can be manufactured in thin layers, which makes them suitable for use as prefabricated crowns and occlusal splints, because the shapes are less bulky and approach anatomical morphology. Combined with the tooth-like shade, the appearance of polycarbonate splints is more esthetic compared to the commonly used transparent PMMA, which increases patient compliance [18].

In this study, the release of BPA from polycarbonate crowns was tested in two extraction media. The artificial saliva was representative of the oral environment, whereas methanol was used for the simulation of the worst-case scenario of BPA release, as polycarbonates are hydrophobic and thus release higher amounts of various components in organic than in aqueous media [12]. This premise was confirmed by the present results, which revealed that the release of BPA in methanol was significantly higher compared to the artificial saliva (Table 1), leading to the rejection of the first null hypothesis. The second null hypothesis had to be rejected as well because the rate of BPA release decreased significantly after the first day (Table 1, Figure 3 and Figure 4). This is in accordance with previous studies that investigated the kinetics of BPA release from dental composites [22,24,25,26]. However, it should be noted that the average daily release of BPA tended to increase slightly after 3 months compared to 1 month. The increase suggested that polycarbonates could degrade during long-term use.

The comparison of BPA amounts measured in this study with previously published data is problematic due to differences in tested materials, specimen size and shape, manufacturing methods, extraction media, immersion times, and analytical methods. Nevertheless, the amounts of released BPA measured herein were comparable with those reported by Suzuki et al. [15], but higher than values reported by Watanabe [16,17]. They were also considerably higher than in recent studies that investigated the release of BPA from dental composites in methanol [22], artificial saliva [24], and distilled water [27].

To estimate the risk associated with the use of dental polycarbonates, the amounts of BPA released during the first day were compared with the standard daily exposure of 1.449 μg/kg b.w. and the TDI of 4 μg/kg b.w. proposed by EFSA [5]. As the released amounts ranged from 8.0 ± 1.6 μg/g to 32.2 ± 3.8 μg/g in methanol and from 0.07 ± 0.02 μg/g to 7.1 ± 0.9 μg/g in the artificial saliva, a single crown (mass 0.11–0.13 g) represents just a minor addition to the standard daily exposure to BPA, which remains well below TDI. However, the use of occlusal splints covering an entire dental arch (mass up to 3 g) could—in the worst-case scenario—equal the standard daily exposure for a 70-kg man or approximately 50% of TDI in individuals weighing 50 kg. While these calculations only apply to the first-day extraction from TBP in methanol, they indicate that polycarbonate occlusal splints could be a relevant source of BPA. Clinically, the exposure might be increased by salivary enzymes, pH and thermal changes, mechanical loading, and other factors, but on the other hand, the exposed surface of the splints would be lower compared to this in vitro study, thus releasing less BPA. To limit the release of BPA, we suggest immersion of the splints in water for at least 1 day before their delivery to the patient, to take advantage of the rapid initial release of BPA. While the release of BPA would be faster in organic media such as alcohols and their aqueous mixtures, they could induce degradation of polycarbonates and affect their mechanical properties, so further research is necessary to support this alternative.

The comparison between polycarbonate materials showed that the lowest amounts of BPA were released from prefabricated crowns. The observed smooth surface (Figure 5A) and parting line (Figure 6A) indicated that the crowns were presumably manufactured by injection or compression molding. In contrast, milled ZPF and TBP crowns exhibited the highest release of BPA after 1 day in both methanol and the artificial saliva. This could be related to the presence of plastically deformed areas and cracks, especially on inner surfaces (Figure 5B,C), which increased contact with the extraction media [12]. Moreover, heat generated during milling could elevate surface temperature. As a result, the polycarbonate could become more susceptible to hydrolytic degradation and release more BPA [28,29,30]. ZPF was processed according to the manufacturer’s instructions, i.e., using a single edge milling bur, which allows for effective chip removal and prevents overheating of the material [18]. In contrast, the manufacturing process recommended for TBP involves a bur with two cutting edges and a higher rotation speed, which could generate more heat. Water cooling is therefore required for TBP, and we assume that it could lead to increased hydrolytic degradation and cause the significantly higher release of BPA from TBP compared to ZPF, which was milled in the dry milling mode with air cooling. There might also be a difference in the quality of the blanks between ZPF and TBP, but the manufacturers did not provide any details about their composition and manufacturing conditions.

The 3D-printed crowns initially released less BPA than milled crowns, possibly due to fewer defects on the surface. However, their release of BPA did not decrease as markedly after the first day and exceeded that of milled polycarbonates in methanol after 1 week, 1 month, and 3 months. The different kinetics of BPA release and the highest sorption of the artificial saliva among polycarbonates might indicate that the bulk properties of 3D-printed crowns were adversely affected by the heating of the filament to approximately 280–300 °C during printing. This is because polycarbonates absorb air humidity, which could trigger hydrolytic degradation at such high temperatures [30,31]. While similar temperatures are usually used for molding, which was presumably used for the manufacturing of prefabricated crowns, degradation is limited by the thorough drying of the polycarbonate pellets and their processing under controlled conditions [30].

The sorption of 3D-printed crowns (15.5 ± 1.8 µg/mm^3^) was in accordance with a previous report on 3D-printed splints [32]. Other polycarbonates exhibited significantly lower values of the artificial saliva sorption. Their values ranged from 2.7 to 3.9 µg/mm^3^ which corresponded to 0.24–0.33 wt%. This was significantly higher compared to the water sorption of a polycarbonate temporary crown (0.07 wt%) reported by Watanabe [17], but similar to other reports on polycarbonates [30,33]. The measured amount of extractable matter in the artificial saliva was 1.7–3.2 µg/mm^3^ (0.14–0.29 wt%), which is in accordance with previously reported values [30,32].

As polycarbonates are a clinical alternative to PMMA, it was used as a control in this study. Trace amounts of BPA were detected in methanol during the first week, possibly as a result of contamination during blank manufacturing or milling. In the remaining extracts, BPA was not detected, meaning that its content was lower than LLOQ. The sorption of PMMA in the artificial saliva was approximately five times higher compared to milled polycarbonates, which corroborates a previous study on the water sorption of injection-molded thermoplastic denture base materials [33]. The amount of extractable matter of PMMA was similar to polycarbonates. The immersion in methanol led to the swelling and plasticization of PMMA crowns, which lost their original shape after approximately 1 week. No such changes were observed in the artificial saliva or with polycarbonate crowns in either of the media.

## 5. Conclusions

Within the limitations of this in vitro study, we conclude that dental polycarbonates could be a relevant source of BPA when used as occlusal splints. However, the tolerable daily intake was not exceeded even in the worst-case scenario simulated by immersion in methanol, where significantly more BPA was released compared to storage in the artificial saliva.

## Figures and Tables

**Table 1:** The average daily release of BPA in μg/g (Mean ± SD).

Material	1 Day (Day 1)	1 Week (Days 2–7)	1 Month (Days 8–28)	3 Months (Days 29–84)
	**Methanol**			
Prefabricated polycarbonate crowns	8.0 ± 1.6 bA *	1.2 ± 0.2 bB *	0.80 ± 0.11 bC *	1.2 ± 0.1 bB *
Milled Zirkonzahn Temp Premium Flexible (ZPF)	22.8 ± 7.7 cA *	2.1 ± 0.3 cB *	1.1 ± 0.2 bcC *	1.2 ± 0.06 bC *
Milled Tizian Blank Polycarbonate (TBP)	32.2 ± 3.8 dA *	4.0 ± 0.3 dB *	1.3 ± 0.2 cC *	1.7 ± 0.3 cD *
3D-printed crowns	11.1 ± 2.3 bA *	7.2 ± 1.6 eB *	1.4 ± 0.2 cC *	1.9 ± 0.2 cD *
Milled Zirkonzahn Temp Basic (PMMA)	0.04 ± 0.03 aA *	0.02 ± 0.01 aA *	0.00 ± 0.00 aB	0.00 ± 0.00 aB
	**Artificial Saliva**			
Prefabricated polycarbonate crowns	0.07 ± 0.02 bA *	0.01 ± 0.00 bB *	0.02 ± 0.00 bBC *	0.03 ± 0.00 bC *
Milled Zirkonzahn Temp Premium Flexible (ZPF)	0.34 ± 0.03 cA *	0.11 ± 0.01 cB *	0.11 ± 0.01 dB *	0.18 ± 0.01 dC *
Milled Tizian Blank Polycarbonate (TBP)	7.1 ± 0.9 dA *	1.1 ± 0.1 dB *	0.58 ± 0.04 eC *	0.59 ± 0.05 eC *
3D-printed crowns	0.12 ± 0.13 bA *	0.10 ± 0.07 cA *	0.06 ± 0.02 cA *	0.07 ± 0.02 cA *
Milled Zirkonzahn Temp Basic (PMMA)	0.00 ± 0.00 aA *	0.00 ± 0.00 aA *	0.00 ± 0.00 aA	0.00 ± 0.00 aA

Different lowercase letters indicate statistically significant differences between materials for each extraction medium, different uppercase letters indicate statistically significant differences between immersion times (*p* < 0.05). * indicate statistically significant differences between amounts of BPA released in methanol and the artificial saliva for each material (*p* < 0.05).

**Table 2:** Sorption and amount of extractable matter in the artificial saliva in µg/mm^3^ (Mean ± SD).

Material	Sorption	Amount of Extractable Matter
Prefabricated polycarbonate crowns	3.9 ± 0.8 b	1.7 ± 1.0 ab
Milled Zirkonzahn Temp Premium Flexible (ZPF)	2.7 ± 0.5 a	3.2 ± 0.5 b
Milled Tizian Blank Polycarbonate (TBP)	2.8 ± 1.0 ab	2.9 ± 1.2 ab
3D-printed crowns	15.5 ± 1.8 c	1.3 ± 1.0 a
Milled Zirkonzahn Temp Basic (PMMA)	17.2 ± 0.8 c	2.7 ± 0.9 ab

Different lowercase letters indicate statistically significant differences between materials (*p* < 0.05).

## Data Availability

Data available on request from the corresponding author.
